# Increased cortical reactivity to repeated tones at 8 months in infants with later ASD

**DOI:** 10.1038/s41398-019-0393-x

**Published:** 2019-01-30

**Authors:** Anna Kolesnik, Jannath Begum Ali, Teodora Gliga, Jeanne Guiraud, Tony Charman, Mark H. Johnson, Emily J. H. Jones, The BASIS Team

**Affiliations:** 1 Centre for Brain and Cognitive Development, Birkbeck, University of London, London, UK; 2 Core CAMHS (Child and Adolescent Mental Health Service), Brookside Family Consultation Clinic, Cambridge, UK; 3 Psychology Department, Institute of Psychiatry, Psychology & Neuroscience, King’s College London, London, UK; 4 Department of Psychology, University of Cambridge, Cambridge, UK

## Abstract

Dysregulation of cortical excitation/inhibition (E/I) has been proposed as a neuropathological mechanism underlying core symptoms of autism spectrum disorder (ASD). Determining whether dysregulated E/I could contribute to the *emergence* of behavioural symptoms of ASD requires evidence from human infants prior to diagnosis. In this prospective longitudinal study, we examine differences in neural responses to auditory repetition in infants later diagnosed with ASD. Eight-month-old infants with (high-risk: *n* *=* 116) and without (low-risk: *n* *=* 27) an older sibling with ASD were tested in a non-linguistic auditory oddball paradigm. Relative to high-risk infants with typical development (*n* = 44), infants with later ASD (*n* = 14) showed reduced repetition suppression of 40–60 Hz evoked gamma and significantly greater 10–20 Hz inter-trial coherence (ITC) for repeated tones. Reduced repetition suppression of cortical gamma and increased phase-locking to repeated tones are consistent with cortical hyper-reactivity, which could in turn reflect disturbed E/I balance. Across the whole high-risk sample, a combined index of cortical reactivity (cortical gamma amplitude and ITC) was dimensionally associated with reduced growth in language skills between 8 months and 3 years, as well as elevated levels of parent-rated social communication symptoms at 3 years. Our data show that cortical ‘hyper-reactivity’ may precede the onset of behavioural traits of ASD in development, potentially affecting experience-dependent specialisation of the developing brain.

## Introduction

Autism spectrum disorder (ASD) is defined by difficulties in social communication, as well as the presence of restricted interests, repetitive behaviours, and sensory anomalies^1^. Symptoms emerge in the first years of life, and can be reliably identified through behavioural assessments from toddlerhood. Several recent theories have implicated dysregulated coordination of excitatory and inhibitory signals (E/I) in cortical processing and associated homoeostatic/autoregulatory feedback loops as one potential common mechanism through which multiple background genetic and environmental risk factors could converge to produce behavioural symptoms of autism^2–7^. The high co-occurrence of epilepsy in individuals with ASD (22%) forms one line of evidence for this hypothesis^8^, in addition to the high rates of ASD in genetic disorders that disturb GABA-ergic functioning (the primary source of inhibitory signalling in the brain), including Fragile X, 15q11-13 and Neurofibromatosis Type 1^9–11^. Alterations in GABA and glutamate levels have been identified in adults and children with ASD using magnetic resonance spectroscopy (MRS)^12–14^, although findings are inconsistent across studies and are limited by differences in the measurement variables selected and the lack of spatial precision inherent to this measurement technique. In animal models of ASD, emerging evidence from stem cell studies have implicated an over-production of GABA-ergic neurons^15^, while several animal models provide support for both GABA and glutamatergic dysfunction in ASD-related phenotypes^7,16–18^. Multiple genetic and environmental risk factors for ASD may converge to disrupt the coordination between excitatory and inhibitory neurotransmitters in the developing brain.

Although there is a reasonable body of evidence linking dysregulated E/I to ASD, establishing a causal relationship to symptoms requires mapping these disruptions before symptoms emerge. Genetic evidence indicates that risk factors for alterations in E/I balance are expressed prenatally, including mutations in genes involved in synaptic development and function^6,7,19^. Full implications for early brain development remain unclear, particularly since excitatory and/or inhibitory signalling is dynamically shaped over development^20,21^. For example, GABA-ergic signals are initially excitatory before shifting to their mature inhibitory function sometime in late prenatal/early postnatal development^22^. Considerable homoeostatic pressure coordinates developmental changes in E/I coordination^23,24^, and activity-dependent GABA signalling is thought to be critical in optimising the balance between excitation and inhibition in the developing cortex^25^, with a critical role in shaping cortical sensitive periods. The importance of the interplay between E/I signalling is further supported by the finding that the Nrg1 and ErbB4 excitatory signalling pathways control development of inhibitory circuitry in the mammal cerebral cortex by regulating connectivity of GABA-ergic neurons^26^. Dysregulation within this pathway has been associated with altered plasticity and the emergence of neurodevelopmental disorders such as schizophrenia and ASD^27,28^. Early alterations in E/I coordination will be influenced by substantial homoeostatic, autoregulatory or adaptive processes that may further compound initial perturbations and alter phenotypic expression over developmental time^3,5,29^. Understanding the nature of these changes in ASD will require direct evidence of the presence and consequences of altered E/I balance in human infants prior to symptom onset. The first year of life may be a critical window of interest, since evidence from analysis of SCN2A mutations indicates that over-excitation specific to the first year can result in later autism^30–32^. Considering the availability of pharmacological manipulations that may act on E/I function^16^, studying this system before the age of 1 year also holds promise for effective delivery of pre-emptive interventions^16,33^. To do this, researchers need putative indices of the effects of dysregulated E/I signalling on cortical processing that can be measured in human infants.

One such candidate is electrical activity of the cortex measured during a stimulus repetition paradigm. Repetition of a stimulus typically produces a dampening in subsequent neural responses to that stimulus—a phenomenon termed ‘repetition suppression’ that has been linked to neurotransmitter systems important in inhibitory control^34–36^. For example, blocking GABA activity in the inferior colliculus causes more neurons to respond to repeated stimuli^37^; and activation of GABAergic receptors in the medial geniculate body decreases responses to common stimuli^38^. Further, models of Fragile X have revealed network hyper-excitability in response to repeated auditory stimulation, including decreased glutamatergic drive on GABAergic inhibitory neurons in sensory cortex^10,39^. Thus, examining repetition suppression can provide us with insight into inhibition/excitation balance in the developing brain. In young infants, repetition suppression can be readily measured using EEG^40,41^ a methodology optimal for use with nonverbal populations^33^.

Emerging evidence suggests that repetition suppression may be altered in the early development of ASD. Indeed, there is broad evidence of reduced repetition suppression or habituation in the visual, auditory, and tactile domains of individuals with ASD relative to typically developing infants and children^36,42–45^. Alterations may be particularly pronounced in the auditory domain (indeed, GABA levels measured through MRS are most consistently atypical in the auditory cortex^12,46,47^). For example, Orekhova et al. linked reduced auditory gating of early-stage event-related potential (ERP) (P50) responses^48^ to higher ongoing gamma power in 8–12-year-olds with ASD^49^. Others reported atypical gamma activity during auditory processing in children with ASD and first-degree relatives^50,51^ as well as atypical theta oscillations in response to speech in adolescents and adults with ASD^52^. Alterations in auditory repetition suppression have also been reported in adolescents and adults with Fragile X, a genetic condition where 50% of individuals also meet the criteria for ASD and in which there is robust evidence of increased excitatory activity^53^. Specifically, the researchers found elevated baseline (pre-stimulus) gamma power in response to a tone repetition paradigm, which was associated with reduced habituation of ERPs. Further, there was a concomitant elevation of phase-locking in alpha/beta EEG activity to repeated tones, which was interpreted as a further indication of over-responsiveness in cortical networks. Preliminary evidence also indicates altered EEG responses to auditory or multimodal stimuli in infants at familial risk for autism^40,43,44,54^, including an initial report of decreased habituation of responses to auditory tones^55^. Taken together, examining neural indices of repetition suppression in the infant brain may provide an index of cortical hyper-reactivity that is sensitive to early alterations in E/I coordination.

### The present study

In the present study, we examined neural responses to repeated auditory tones in an oddball paradigm conducted with 8-month-old infants at low (*n* = 27) and high (*n* = 116) familial risk for ASD, an age prior to diagnosis. Additionally, stimulus-locked gamma-band activity may become easier to detect in typical development within the first year of life^56,57^. Infants at high familial risk for ASD have a 20% chance of developing ASD themselves^58^, and thus longitudinal studies of this population allow examination of causal paths to the disorder. Previously reported ERP data from studies of auditory processing (including a small subset of infants also included in the present investigation) showed no dampening of response following repetition in infants at high risk^55^, although no ASD outcome was available at the time. In the present study, we analysed the underlying spectrogram by breaking down the signal into different frequency bands^59^, which reveals more specific information about the functional organisation of brain activity within the same study design as classic ERP paradigms. Two features of the EEG signal that were expected to produce a robust change with stimulus repetition in the neurotypical brain were evoked high-frequency gamma and inter-trial phase-coherence in the alpha/beta band^60,61^. Both indices have been linked to E/I balance in previous work^53,54^. We focused on evoked/time-locked, and not induced activity, because these signals are easier to separate from some of the non-time-locked artefacts that are common in the analysis of ongoing EEG (such as motion, myogenic artefact or electrical line-noise).

As we were concerned about multiple comparisons and the likelihood that effects may be subtle, we took two a priori analytic decisions. First, because the previous literature did not provide clear guidance for a priori selection, we selected time windows, AOIs and frequency bands based on data from the low-risk infants. Specifically, we identified bands/areas associated with habituation of gamma responses across repeated tones in the low-risk group. We then restricted the analysis of the high-risk group to those bands/areas. This avoids multiple comparisons within the high-risk analysis, but does increase the possibility that there are group differences in other scalp regions that we did not assess. Post-hoc analyses conducted to mitigate this are included in the SM. For the ITC, we analysed activity over the same region of interest but selected the time-window and frequency band based on both previous literature and the aggregated grand average technique. Second, two approaches to group analysis have been traditionally used in the infant sibling literature. One is to compare indices across four ‘outcome’ groups: low risk, high-risk typical development, high-risk atypical development or high-risk ASD. We did not follow this path because we had already used the low-risk group to identify windows of interest (and thus including them in comparisons was unfair). Also, the intermediate HR-Atyp group was excluded from main analysis as (1) it would reduce power and (2) no clear predictions could be made about this group (for analysis including the HR-Atyp group, see SM6). The second strategy is to compare responses between high-risk infants with typical development (HR-TD) and those with ASD (HR-ASD), which is the most faithful design to the case/control approach used in the literature^58,62^. This approach was used in the present manuscript. It was predicted that there would be reduced or absent repetition suppression response in the gamma band as well as elevated phase-coherence to repeated tones in the ASD group relative to the high-risk typically developing group^53,55,63^.

To exploit the full dimensional nature of the high-risk design, we then examined whether a *cortical reactivity index* (CRI) (a composite of gamma habituation and ITC) was associated with dimensional variation in later language skills across the whole cohort (LR, HR-TD, HR-Atyp, HR-ASD^45,64^). The term ‘reactivity index’ was selected to represent the conceptual nature of these EEG responses. Specifically, when a repeated train of stimuli are encountered, ongoing oscillatory activity in the brain can change in two ways. The power or amplitude of oscillations can increase in response to the stimulus; and/or the phase of the oscillation can align with the timing of the stimulus in a consistent way across trials^65^. Both these reactions produce larger amplitude event-related brain responses and thus in a conceptual sense reflect reactivity. Thus, reduced gamma habituation and higher ITC would additively work to produce a higher CRI. Given the critical role of E/I coordination in experience-dependent specialisation^66,67^, it was predicted that alterations in neural responses to auditory tones should be associated with poorer language outcomes, as this is a key skill that develops through experience-dependent tuning to the sounds and patterns of the child’s language environment^68,69^. Finally, it was expected that higher scores on the index would be positively associated with parent-report measures of ASD-related traits at 3 years^70^.

## Methods

### Participants

Participants were 116 high-risk (HR) (64 male; 52 female) and 27 low-risk (LR) (14 male; 13 female) children. ‘High-risk’ infants had an older full sibling with a community clinical diagnosis of ASD (recruited from the British Autism Study of Infant Siblings, BASIS—Phase 2; http://www.basisnetwork.org). Infants in the ‘low-risk’ group had no reported family history of ASD or other developmental or genetic disorders (recruited from a volunteer database at Birkbeck Centre for Brain and Cognitive Development), and had at least one older full sibling. Infants recruited for the study attended four visits, at 8 months and 14 months, with follow-up visits at 2 and 3 years. EEG data for the present study are taken from infants during their 8-month visit (*Mean* = 9.03m, *Standard Deviation* *=* 1.1m) and outcome data from the 3-year visit (*Mean* *=* 39.05m, *Standard Deviation* = 3.47m). The study was approved by the National Research Ethics Service London Central Ethical Committee (08/H0718/76) and conducted in accordance with the Declaration of Helsinki (1964). Further details of inclusion/exclusion criteria and proband diagnostic phenotyping are provided in SM1: Clinical Assessment.

Within the high-risk group, developmental outcome at 3 years was used as a grouping variable (See Supplementary Materials SM1 for details of assessment). Sixty-four infants were considered typically developing and constituted the HR-TD group. Seventeen infant siblings met gold-standard criteria for ASD (determined by consensus clinical judgement of a group of expert clinical researchers based on information including the ADOS-2, ADI-R and interaction with the child; see SM1, ST2). Lastly, 32 infant siblings were classified as atypically developing, i.e. displayed some developmental concerns but not meeting criteria for an ASD diagnosis (HR-Atyp). Following data cleaning procedures, 131 participants provided sufficient EEG data for analysis (*N* = 94/113*;* LR: 14/27; HR-TD = 45/64; HR-Atyp = 21/32; HR-ASD = 14/17; see Table ST1). Sensitivity analysis^71^ of the total sample size with a power of 1−*β* = .80 revealed a population effect size of *d* *=* .21.

### Stimuli

Sounds were presented in an oddball paradigm originally designed by Guiraud et al.^55,72^. Duration of the sound was 100 ms, with 5 ms rise and fall time. The inter-trial interval was 700 ms. A ‘Standard’ pure tone at 500 Hz was presented with a 77% probability. The paradigm also included two deviant or infrequent tones, which were presented with a 11.5% probability each. One infrequent sound was a white noise deviant, while the other was a pure tone of 650 Hz (pitch deviant). The sound intensity was 70 dB SPL. The sounds were presented for 5–7 min or until the infant became too restless, which on average yielded 477 trials for low-risk and 462 trials for high-risk infants (See Table ST1 for trial breakdown). Following Guiraud et al.^55^ and a priori hypotheses, responses to the first, second, and third presentation of a Standard tone were examined.

### Procedure

The auditory oddball task was administered at the end of a battery of visual EEG tasks. Infants were seated on the parent’s lap facing the experimenter, who blew soap bubbles throughout the recording session to keep the infant calm and engaged. The experiment was conducted in a sound attenuated room, where the sounds were presented from two speakers, 1 m apart, and located 1 m in front of the infant. The Mullen Scales of Early Learning^73^ were administered in the standardised format; with assessments completed in the same laboratory setting by a small team of experimenters. Parents were also asked to complete a set of questionnaires at home at each visit, including the Social Responsiveness Scale^70^ at 3 years of age.

### EEG recording and pre-processing

Electrophysiological activity was measured using an EGI 128-electrode Hydrocel Sensor Net with the vertex electrode as reference and sampled at 500 Hz. A 0.1–100 Hz band-pass filter was applied offline. The recording was segmented into 1000 ms sections (500 ms pre- and 500 ms post-stimulus presentation). Bad channels in each segment were marked by automatic artefact detection and visual inspection in NetStation (v. 4.5.6). The segments with pronounced artefacts, i.e. gross motor movement, eye blinks, or more than 25 bad channels, were rejected from analysis (104 clean data sets remained). All epochs exceeding 150 μV at any electrode were excluded. At least 30% of trials had to be retained in each category to qualify the dataset to be included in the group analysis. For the remaining trials, channels with a noisy signal were interpolated from neighbouring channels with a clean signal using spline interpolation. Following Guiraud et al.^55^ and Seery et al.^45^, all standards that followed either deviant were categorised by position—i.e. Standard 1, Standard 2, and Standard 3, to which the present analysis is confined. There was only one restriction during stimulus presentation—that the deviant sound had to follow at least two standard tones. Due to this, there were notably fewer instances of S3 than S1 and S2. The final number of remaining, artefact free, trials did not significantly differ by group (all *p*s > .05, see ST1).

### Wavelet transform

Four regions of interest (ROIs) in the left and right frontal and tempo-parietal cortex were chosen based on previous investigations into auditory gamma activity^24,25^ (S1). One hundred and four pre-processed data sets were exported into MatLab® using the free toolbox EEGLAB (v.13.6.5b, http://sccn.ucsd.edu/eeglab/) and re-referenced to the average reference. For analysis of evoked gamma, epochs of raw EEG data were averaged together. A custom-made collection of scripts, WTools (available upon request from Dr Eugenio Parise (available from Dr Parise via email: eugenioparise@tiscali.it)), was used to compute complex Morlet wavelets at 1 Hz intervals between 1 and 80 Hz. A continuous transformation was applied to all epochs through convolution with a wavelet at each frequency in the chosen range, taking the absolute value as a result (i.e. amplitude not power^75^). To reduce distortion created by convolution, padding of 100 ms at the start and end of the segment was applied to the individual data sets. A baseline period was set between −200 and 0 ms and subtracted from the post-stimulus responses to remove any residual 50 Hz (electrical) noise in the data and to control for pre-stimulus preparatory activity. Amplitude was extracted for low (20–40 Hz) and high (40–60 Hz) gamma bands (30–150 ms post-stimulus onset). The low and high bands were derived from previous examinations of gamma-band activity in infants^57^. Such evoked (rather than induced or baseline) gamma reflects responses time-locked to the stimulus, reducing the likelihood of contamination by muscle artefact or electrical noise.

Secondly, ITC measures were calculated (collapsed across all standards to improve signal-to-noise ratio). This measure represents whether the distribution of phase angles in a single time-frequency point is uniform, with 1 = perfect phase consistency across trials and 0 = c completely random phase angles^60,76,77^. When extracting ITC, small trial numbers can artificially skew results^76^. To identify whether a phase-locking response occurred, we collapsed across all standards for this analysis, such that there were at least 100 good trials included per infant. Individual number of trials per data set were entered as covariates in the model, which also reduced the possibility of introducing further biases from selecting a fixed subset of trials. The number of trials did not affect the stability of the oscillatory response, where the common cut-off for the number of trials used ranges from one^78^ to between 200 and 800^79,80^, with many studies not reporting these values^81,82^. Average ITC was extracted using EEGLAB functions in the 10–20 Hz band, with this frequency band and time-window selected based on the aggregated grand average, previous work with individuals with Fragile X^53^ and the onset timing of the P150 infant ERP component, which has been shown to be sensitive to reduced habituation in a subset of the high-risk infants (100 to 180 ms; Figure S2)^55^. However, because ITC is also commonly measured in the theta range in previous studies of auditory processing in infancy, analysis of ITC in the 3–6 Hz is also included in the SM (SM5.2. ITC analysis 3–6 Hz).

Analysis of the P150 component response has been conducted and visualised separately in the SM^40,45,55^ (details in Figure S2, Supplementary Materials). ERPs are not discussed in the main text beyond this point given that they represent an unspecified combination of power and phase changes in oscillatory rhythms. On the other hand, time-frequency analysis directly relates to the hypothesis under investigation and provides greater sensitivity revealing differences in auditory perception in the developing brain^83^.

### Analysis strategy

The analysis approach was designed to constrain the number of contrasts made in testing effects of developmental outcome, in order to minimise Type 1 error and maximise power in this relatively modest sample. Accordingly, gamma responses to tone repetition were first analysed within the low-risk group to select the topography and frequency band associated with a normative repetition suppression (RS) response. RS was defined as a reduction in gamma amplitude between the first and third standards, given that oscillatory response asymptotes after a second repetition^84^. We then focused on that region and frequency band, contrasting responses between the HR-TD and HR-ASD groups^85,86^. Greenhouse−Geisser corrections were applied where appropriate. Similarly, we investigated phase-locking by comparing ITC over the scalp region used in the analysis for HR-ASD and HR-TD groups. The HR-Atyp group were excluded from these analyses for greatest comparability with work with older children using case/control designs^85,86^; and again to maximise power given the relatively modest sample. However, their data are presented in SM6 (in addition to broadly consistent analyses presented using the alternative approach of pooling the high-risk infants with typical and atypical development into ‘HR-noASD’ groups).

Repetition suppression of evoked gamma and low-frequency ITC were then used to create a ‘Cortical Reactivity Index’ by z-scoring each measure across the cohort (lower repetition suppression of gamma between first and third Standard and greater ITC would give a higher index score), and then averaging the values. We then examined effects of group and continuous relations with dimensional outcomes across the whole cohort. These included development in language skills (difference scores of Expressive and Receptive Language subscales, calculated by subtracting 8-mo from 36-mo scores)^73^, ADOS severity scores^87^ at 36 months, as well as the *t*-score total on the Social Responsiveness Scale™ (SRS™^57^) at 36 months.

## Results

### Repetition suppression analysis

#### Low risk (20–40 Hz, 40–60 Hz)

A paired-samples *t* test was carried out to examine reductions in amplitude of evoked gamma between the first and third repetition of each standard (1st vs. 3rd Standard) over left and right tempo-parietal regions in the two frequency bands respectively. This indicated a significant decrease in high (40–60 Hz) gamma amplitude (repetition suppression) over the right tempo-parietal region [*t*(13) = 2.58, *p* = .023, *η*^2^ = .35] but not the left [*t*(13) = −.65, *p* = .527, *η*^2^ = .034]; with no significant differences in the 20–40 Hz band (*t*s < 1.6, *p*s > .54). Corresponding analyses over frontal ROIs revealed no significant effects across either gamma band (*t*s < 1, *p*s > .21).

#### High-risk (40–60 Hz)

**Fig. 1:**
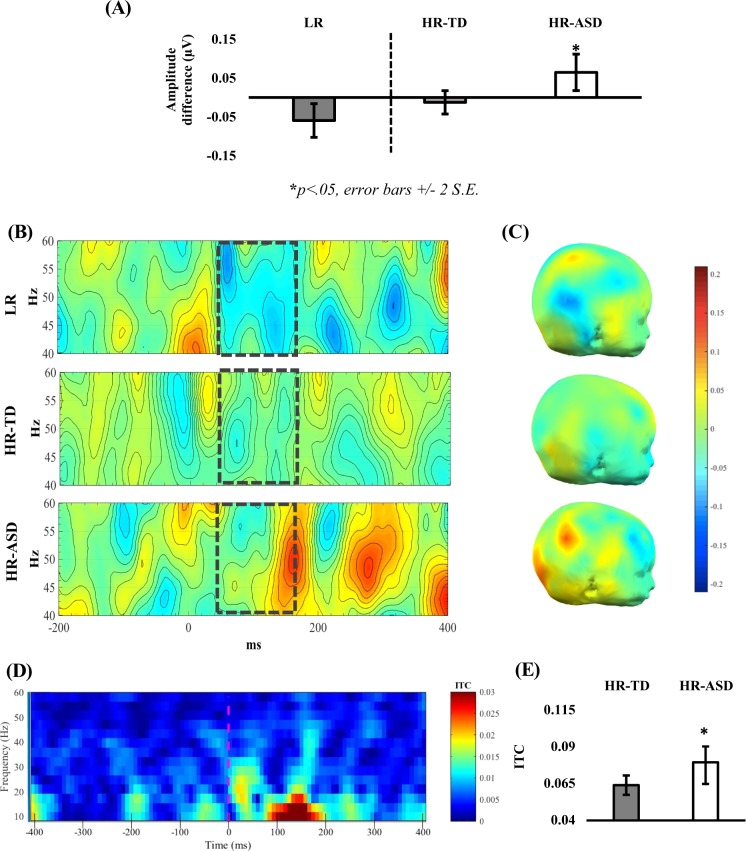
Gamma and ITC plots for LR, HR-TD and HR-ASD groups. **a** Amplitude difference of 40–60 Hz evoked gamma in the right tempo-parietal electrodes between Standard 3 and Standard 1. **b** Difference plots of the gamma responses to the repetition of standard tone (Standard3 − Standard1) in 40–60 Hz evoked gamma in right tempo-parietal electrodes. It appears while the LR group shows a clear repetition suppression, the HR-ASD group shows an increase in gamma activation. **c** 3D Scalp maps of the difference in total 40–60 Hz gamma activation between third and first frequent tone in the 100–150 ms time period in the three groups. **d** Total inter-trial coherence responses collapsed across all infants and all Standard trials. The 100−180 ms time-window and 10–20 Hz band was chosen as the area of interest for group comparisons. **e** Inter-trial coherence values for all standards collapsed together in the high-risk infant siblings from right tempo-parietal scalp region. LR group included for reference. Note. Error bars depict standard error of the mean for each group respectively. LR low risk. * Denotes signficance level (i.e. *p* < .05)

Based on the pattern of findings in LR infants, analysis of ASD outcome was constrained to the 40−60 Hz frequency band over the right tempo-parietal region. A one-way ANOVA revealed an effect of group on Standard 3 – Standard 1 difference scores [*F*(1,55) = 6.67, *p* = .012, *η*^2^ = .105]; which remained significant after co-varying trial numbers [*F*(1,55) = 6.53, *p* = .013, *η*^2^ = .105]. Specifically, there was a decrease in gamma activation between the first and third repetition in HR-TD, relative to an increase in the HR-ASD group. Figure 1 illustrates the responses for the three groups; SM3 indicates that responses were not due to ocular muscle activity^88^ (see SM3). Notably, the main effect of group persisted when the HR-Atyp group was included in the model (see SM5). Our additional post-hoc analyses of evoked theta (3–6 Hz over 50–400 ms) and late evoked gamma (40–60 Hz over 200–350 ms) did not reveal significant differences for high-risk groups (SM5).

#### ITC

Within the same ROI (right tempo-parietal region), a univariate ANOVA was used to examine group differences (HR-ASD, HR-TD) in ITC in the alpha-beta band (10–20 Hz). This revealed significantly *greater* ITC in the HR-ASD than the HR-TD group [*F*(1,55) = 4.62, *p* = .036, *η*^2^ = .06]; which remained significant when co-varied with the number of trials infants were presented with [*F*(1,54) = 4.58, *p* = .037, *η*^2^ = .06]. When the HR-Atyp group was included, there were no significant differences in ITC across the three risk groups (see SM6 for further details of this exploratory analysis). Additional post-hoc analysis of ITC within the theta range revealed similar phase-locking values in the same time period, with no significant differences in phase-locking values between the high-risk groups (all *p*s > .2; SM5.2).

#### Cortical reactivity index

A composite CRI was created by computing *z*-scores for the 40–60 Hz evoked gamma and 10–20 Hz ITC responses for the whole high-risk group, and then averaged across the two indices. A higher score on the index would reflect diminished auditory repetition suppression. As expected, an ANOVA indicated significantly higher scores in the HR-ASD than the HR-TD group [*F*(1,55) = 15.16, *p* < .001, *η*^2^ = .22]. A logistic regression indicated that this index correctly classified 93% of the HR-TD group and 50% of the ASD group [*β* = 2.74, S.E. = 0.87, *w* = 9.89, *p* = .002]. When the whole sample was included, the main effect of group remained significant, and HR-ASD infants maintained significantly higher CRI scores than the other groups (*p*s between .002 and .045; see SM4).

**Fig. 2:**
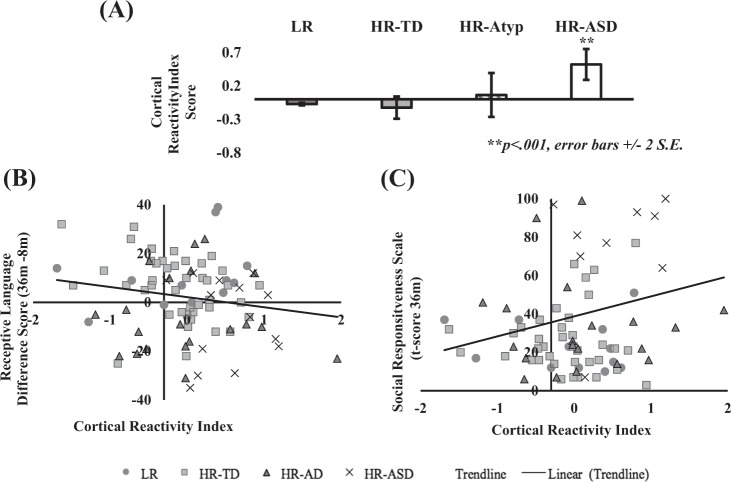
Associations between cortical reactivity index scores and behavioural data. **a** Cortical reactivity index *z*-scores for all outcome groups. Note that the HR-Atyp group is visualised here but was not included in the HR-TD vs. HR-ASD comparison. Composite score for difference in evoked gamma (40–60 Hz) and ITC responses (10–20 Hz) over right tempo-parietal ROI was associated with **b** smaller change in Receptive Language scores between 8 and 36 months and **c** higher SRS™ scores, a dimensional measure of ASD-related traits. LR low risk, HR-TD high-risk—typically developing, HR-Atyp high-risk—atypical development, HR-ASD high risk—autism spectrum disorder. Note: The fit line is for an average of all infants. Error bars depict standard error of the mean

As shown in Fig. 2, the composite score was also significantly associated with development in Receptive Language between 8 and 36 months across all infants in the sample [*r*(88) = −0.25, *p* = .032]; controlling for trial number [*r*(88) = −0.25, *p* = .03], and was also associated with SRS™ Total T-scores [*r*(88) = 0.22, *p* = .04]; controlling for number of trials [*r*(88) = 0.22, *p* = .039], but not with change in Expressive Language [*r*(85) = −0.17, *p* = −.20]; controlling for trial number [*r*(85) = −0.12, *p* = .27]. No significant association was found between scores on the index and the ADOS severity scores [*r*(88) = 0.15, *p* = .162]; controlling for trial number [*r*(85) = 0.16, *p* = .135].

## Discussion

Alterations in excitatory and inhibitory signalling are a feature of several leading neurobiological theories of sensory perturbations observed in ASD, with some researchers proposing that behavioural symptomology is the cumulative developmental consequence of altered excitatory and inhibitory coordination within cortical systems^2,3,6,7,89^. To our knowledge, we present the first human evidence that elevated cortical reactivity is present in infants with later ASD prior to the emergence of behavioural symptoms. This is consistent with the presence of alterations in excitatory and inhibitory function in infancy. In our work, reduced repetition suppression of evoked gamma and generally increased alpha/beta phase-locking to auditory repetition were present in 8-month-old infants with later ASD. A combined index of cortical reactivity was associated with reduced receptive language and social communication at 3 years dimensionally across the entire cohort. Altered EEG responses to repetition may therefore be a candidate stratification biomarker for language functioning in ASD. As atypical cortical auditory reactivity has been previously linked to weakened GABA circuits in mouse infancy^67^, our results could reflect a lack of regulation within the E/I balance in infants with later ASD.

Enhanced reactivity within the developing cortex may relate to the alterations in connectivity that have been broadly observed in ASD, due to the experience-dependent nature of synapse development^43,90,91^. Indeed, 14-month-old infants with later ASD and high levels of restricted and repetitive behaviours show significant over-connectivity between frontal and temporal regions^90^. Increased spontaneous activity in early development could also be associated with structural overgrowth^92,93^, because internal activity-driven processes contribute to brain growth^94^. Future work combining both MRI and EEG techniques in young infants will allow us to investigate the bidirectional links between structure and function in the infant brain. Further, longitudinal studies with repeated EEG phenotyping are necessary to determine whether the observed alterations in cortical reactivity represent a developmentally pervasive phenomenon, or a transient developmental delay in the normal increases in inhibitory activity observed in early infancy^2,20^. Several influential theories propose significant mechanisms that would alter the expression of excitatory/inhibitory processing over developmental time^3,7,20^.

Cortical reactivity to repetition of sensory stimuli could reflect processes that disrupt the experience-dependent specialisation of the social brain, as the inhibitory signals critical for repetition suppression are also critical in shaping sensitive periods in early development^16,66^. Altered trajectories of specialisation are an emerging theme from prospective studies of high-risk infants. Across the first year of life infants with later ASD show a decline in interest in eyes and faces^62^, emergence of slowed attention-shifting^95^ and early signs of language delays^33,86^. Such behavioural changes are related to alterations in specialised brain activity. For example, between 5 and 6 months, infants with later ASD show reduced sensitivity over temporal cortex to human sounds^96,97^ and altered ERP responses to pictures of faces^98,99^. Intriguingly, our index of cortical reactivity related to dimensional variation in receptive language growth between 8 and 36 months, an age-range associated with specialisation of language regions^100^. Language acquisition is dependent on attention to novelty and change, and it is likely that poor tuning to repetition would have a detrimental effect on this process. Testing how our index of cortical reactivity relates to more fine-grained indices of language and social communication through a longitudinal investigation will be an important next step.

Elevated cortical reactivity could result from a range of impairments at the molecular level, contributing to reduced inhibition or increased excitation. Select environmental risk factors may have converging effects through oxidative stress, which is thought to impact parvalbumin inhibitory interneurons. This is thought to be a key mechanism for regulation of E/I balance and maintenance of neural microcircuitry^16,101^, and form a unified risk pathway for schizophrenia and some forms of autism^102,103^. This is supported by Selten et al., who provide succinct evidence of cascading effects of the inhibitory system dysregulation in the cortex and hippocampus on signal processing and later phenotypic changes associated with psychiatric and neurodevelopmental disorders^6^. Recent work with knock-out models of ASD (including mice models of Fragile X) also implicates early increases in spontaneous synchronous activity and upregulation of synaptic turnover in sensory cortices as a common phenotype, consistent with excess cortical reactivity^11,104^. In human adults with Fragile X syndrome, reduced habituation of neural responses to tones was associated with elevated gamma at baseline^53^, which in animal models can be rescued with the GABA_B_-receptor agonist arbaclofen^17^. We did not examine baseline or resting state gamma that was previously found atypical in high-risk populations^43,105^ due to the increased likelihood of contamination by muscle artefact, and so pharmacological manipulation studies of the repetition suppression of gamma responses are warranted. Nonetheless, the results provide an index suitable for measuring the effects of novel pharmaceutical treatments on core biochemical pathways implicated in autism.

These results further complement previous findings of altered habituation profiles in ASD. Reduced habituation/repetition suppression of ERP responses have been observed in a subsample of the present cohort of high-risk infants^55^ and repetition of speech sounds in other samples^45^. In the visual domain, toddlers with ASD showed delayed adaptation to repetition, particularly to social stimuli^99^, while adults showed diminished repetition suppression with increasing levels of ASD traits^106^. Repetition suppression is a critical process by which the brain devotes resources to novel and unexpected stimuli. Atypical/reduced repetition suppression is therefore likely to affect the efficacy with which the brain encodes complex stimuli, such as language, which rely heavily on focusing attention on important dimensions of change in auditory signals^64,107^. Atypical repetition suppression could also contribute to exaggerated sensory sensitivities observed in children with ASD^49^, as well as delayed language ability, a strong predictor of developmental outcomes in individuals with ASD^108,109^.

In summary, findings from the current study indicate that cortical reactivity is present at 8 months of age in infants who later develop ASD. It remains to be established whether these responses are stable in early development, or change dynamically over time. This study provides the first evidence for the emergence of cortical consequences within the right tempo-parietal region from alterations in E/I coordination as early as 8 months of age in infants with later ASD, and offers candidate mechanistic pathways by linking alterations in gamma to individual level differences in later language and social functioning.

## Supplementary information

Supplementary Materials
