# Supplementary material for: Increased cortical reactivity to repeated tones at 8 months in infants with later ASD
Source: Transl Psychiatry. 2019 Jan 30;9:46. doi: 10.1038/s41398-019-0393-x (PMC6353960; doi:10.1038/s41398-019-0393-x)
Supplement: Supplementary file 1 — Supplementary Materials [file 41398_2019_393_MOESM1_ESM.docx]

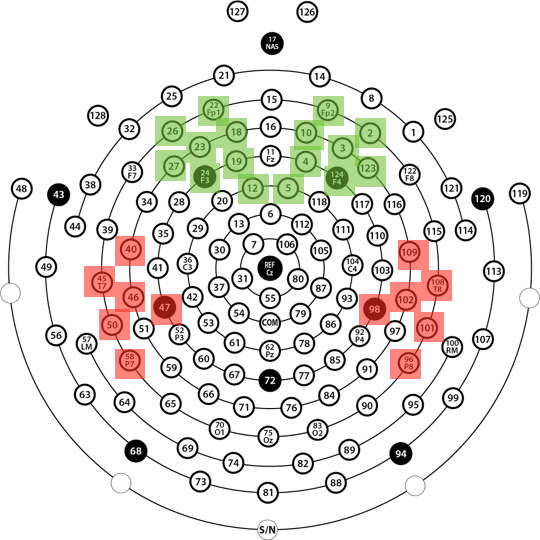


*Figure S1* - Electrode location in four ROIs chosen for analysis. These included averages of Frontal (right 2, 3, 4, 5, 9, 10, 123, 124; left 12, 18, 19, 22, 23, 24, 26, 27) and Tempo-Parietal (right 96, 98, 101, 102, 108, 109; left 40, 45, 46, 47, 50, 58) cites. Selected ROIs were based on previous localizations of ERP and oscillatory responses in similar auditory paradigms (1).

***Table ST1***. *Sample size and descriptive statistics (mean, standard deviation and range) of trials retained for all participants included in the analysis. Total trials depict the number of trials administered.*

| Group | Total Trials | Standard  (Pure tone at 500Hz) | | | | |
| --- | --- | --- | --- | --- | --- | --- |
|  |  | *Total* | *STD1* | *STD2* | *STD3* |  |
| *LR*  *(n =14))* | 467 | 177  (SD = 54.6, 106-261) | 48 (SD=16.29, 24-75) | 48  (SD=17.9, 21-74) | 28.9  (SD=11.6, 12-50) |  |
| *HR-TD*  *(n= 45))* | 468 | 188.6  (SD= 58.04, 108=361) | 50.2 (SD=16.2, 29-91) | 57  (SD=15.4, 31-93) | 34 (SD=10.7, 16-66) |  |
| *HR-Atyp*  *(n = 21))* | 474 | 183  (SD = 70.4, 101-364) | 54.9 (SD=18.4, 26-94) | 57.8 (SD=16.6, 30-90) | 32.9 (SD=9.8, 16-56) |  |
| *HR-ASD*  *(n =14))* | 446 | 211  (SD = 68.2, 97-361) | 48.8, (SD=15.8, 25-76) | 49.4 (SD=17.9, 22-75) | 28.7 (SD=10.2, 13-46 |  |
|  | | | | | |  |
| The number of good trials per participant was entered into a one-way (3 x 4; Condition (Standards 1,2,3) vs. Outcome (LR, HR-ASD, HR-TD, HR-Atyp) ANOVA. It revealed no group differences between the number of trials that were retained for wavelet analysis (all p>.05). Number of trials was also co-varied with the observed habituation effect. The Stimulus order *x* Outcome interaction remained significant when taking into account the trial numbers (*F*(1,57)= 3.99, *p*=.05, *η^2^* =.065). | | | | | |  |

, *p*=.05, *η^2^* =.065).

***SM1. Clinical Assessment***

A battery of clinical research measures was administered to all children at 36 months (see Table 2): the Autism Diagnostic Observation Schedule – Second Edition (ADOS-2;(2)), a standardised interaction observation assessment, was used to assess current symptoms of ASD (116 children were administered Module 2 and 20 children Module 1, ADOS not completed with 5 HR and 2 LR children). Calibrated Severity Scores for Social Affect, and Restricted and Repetitive Behaviours (RRB) were computed (3), which provide standardised autism severity measures, which account for differences in module administered, age and verbal ability. The Autism Diagnostic Interview – Revised (ADI-R; (4), a structured parent interview, was also administered. Standard algorithm scores were computed for Reciprocal Social Interaction (Social), Communication, and Restricted, Repetitive and Stereotyped Behaviours and Interests (RRB). These assessments were conducted without blindness to risk-group status by or under the close supervision of clinical researchers (i.e., psychologists, speech therapists) with demonstrated research-level reliability. Total scores of the Social Communication Questionnaire (SCQ; (5) were used as additional parent report measures of ASD symptoms. The early learning composite scaled score of the Mullen Scales of Early Learning (MSEL; (6) were used to obtain a standardized measure of developmental abilities during testing and the 3 year visit.

Experienced clinicians (TC, GP, CC) reviewed information on ASD symptomatology (ADOS-2, ADI-R, SCQ), adaptive functioning (Vineland-II;(7)), and development (Mullen Scale of Early Learning, MSEL) for each high and low risk child to ascertain ASD diagnostic outcome according to DSM-5 (American Psychiatric Association, 2013). From the 113 HR participants with sufficient EEG data for analysis, 17 (15 boys, 2 girls) met criteria for ASD (hereafter, HR-ASD). From the remaining 96 participants (48 boys, 48 girls), infants were either typically developing (28 boys, 36 girls; HR-TD) or atypically developing (20 boys, 12 girls; HR-Atyp) at 36 months. None of the 25 low-risk children (14 boys, 13 girls; LR) met DSM-5 criteria for ASD and none had a community clinical ASD diagnosis.

For 111 of 116 children with an older sibling with a community clinical diagnosis of ASD (hereafter probands), parents had completed the Development and Wellbeing Assessment (DAWBA; Goodman et al., 2000) and/or the Social Communication Questionnaire (SCQ; Rutter et al., 2003). Seventy-seven probands met criteria on both the DAWBA and SCQ. While a small number scored below threshold on the SCQ (n = 8), no exclusions were made due to meeting threshold on the DAWBA and expert opinion. For 19 probands, confirmation of local clinical diagnosis was only available for the SCQ. For 5 probands, neither measure was available aside from parent-confirmed community clinical ASD diagnosis. Screening for possible ASD in the older siblings of the LR infants was undertaken using the SCQ, with no child scoring above the instrument cut-off for ASD (>15) (one missing). Medical history review confirmed a lack of ASD within first-degree relatives.

***Table ST2***. *Detailed characterization all behavioural assessments completed by the HR subgroups and LR controls at all visits for all participants that contributed data to the present analysis at the 8 and 36months visit.*

|  | ***HR-ASD*** | ***HR-Atyp*** | ***HR-TD*** | ***LR*** |
| --- | --- | --- | --- | --- |
| ***8 months*** |  |  |  |  |
| *Age in months (SD)* | 8.83 (.82) | 8.9 (.69) | 9.10 (.84) | 9.29 (.84) |
| *MSEL Receptive Language* | 47.4 (8.9) | 49.11 (7.3) | 50.98 (9.65) | 49 (11.05) |
| *MSEL Expressive Language* | 55.87 (12.41) | 53 (11.08) | 54.64 (9.4) | 57.64 (9.3) |
| *N (% boys)* | 14 (88.24%) | 21 (65.5%) | 44 (43.75%) | 14 (51.85%) |
|  |  |  |  |  |
| ***36 months*** |  |  |  |  |
| *Age in months (SD)* | 38.56 (1.71) | 38.69 (1.87) | 38.84 (1.60) | 38.72 (1.62) |
| *MSEL Receptive Language* | 39.6 (16.5) | 44.11 (14.78) | 56.91 (9.17) | 59.08 (9.5) |
| *MSEL Expressive Language* | 38.53 (16.05) | 45.63 (12.7) | 57.02 (10.44) | 60.42 (11.3) |
| *SRS™ Total t-scores* | 92.87 (31.82) | 42.14 (30.73) | 29.28 (22.2) | 21.28 (10.34) |
| *ADI-Social* | 12.13 (5.76) | 3.06 (3.16) | 2.00 (2.55) | 0.96 (1.49) |
| *ADI-Communication* | 11.50 (4.69) | 4.44 (4.25) | 2.62 (3.29) | 0.48 (1.05) |
| *ADI-RRB* | 5.63(2.55) | 1.25 (2.15) | 1.49 (2.57) | 2.56 (1.96) |
| *ADOS-Total* | 8.81 (7.68) | 8.13 (4.79) | 2.44 (1.63) | 3.68 (3.09) |
|  |  |  |  |  |

*MSEL – Mullen Scales for Early Learning (t-scores); SRS™ - Social Responsiveness Scale; ADI – Autism Diagnostic Interview; ADOS – Autism Diagnostic Observation Scale (Calibrated Severity Score).*

Individual t-scores on the Expressive and Receptive Scales (6) were entered into a repeated measures ANOVA to look at group differences in age of testing and the scores. The model revealed a significant main effect of Outcome [*F*(1,86)= 10.05, *p*<.001, *η^2^*= .26], as well as an interaction between Age and Outcome [*F*(3,86)=9.01, *p*<.001, *η^2^*= .24]. Means tables suggest that while there was an increase in Expressive and Receptive language scores in the LR and HR-TD groups between 8 and 36 months, infants with later ASD or atypical development showed a worsening in their performance on these scales.

SRS™ total *t-scores* were entered into a one-way ANOVA and significantly differed by group [*F*(3,124)= 33.12, *p*<.001, *η^2^* = .44]. Means tables suggested that infant siblings with later ASD had higher scores on Social Responsiveness Scale (which measures presence and extent of autistic social impairment).

A multi-variate ANOVA was run to look at differences in ADI and ADOS behavioural scales between groups at 36 months. As predicted, all *p* values were under the significance threshold of 0.05, which suggested that the HR-ASD group was rated higher on all symptomatic aspects of ASD-related symptoms relative to the typically developing infants (HR-TD).

***SM2. Event-Related Potentials (P150 analysis)***

ERPs were processed in NetStation 4.5.6. This processing involved filtering the data (0.1-100Hz), visual artefact detection, bad channel replacement, average reference, and baseline correction (-100 to 0ms), which is common practice in the field. They were then averaged by individual, and then by group, and exported to produce the grand average plots (Figure S2). Analysis of the ERP data was based on existent literature (1,8), where infants’ responses to repetition were analyzed using separate mixed-model ANOVA for LR group, and then the high-risk groups (HR-TD vs. HR-ASD) with condition (Standard 1 vs. Standard 3), ROI (frontal vs. tempo-parietal), and hemisphere (right vs. left) as repeated factors, with P150 (average amplitude 150-300ms) as a dependent variable. Our analyses revealed that in the LR group, there was only one main effect of ROI, with significantly higher P150 amplitude in the frontal than tempo-parietal regions [*F*(1,12)= 8.13, *p*=.015, *η^2^* =.4], although this effect disappeared when trial numbers were added as a covariate [*p*=.164]. Due to this, the analysis of risk groups was run for both ROIs and hemispheres. The repeated measures ANOVA showed a main effect of ROI [*F*(1,63)= 102.3, *p*<.001, *η^2^* =.61] and hemisphere [*F*(1,63)=20.39, *p*<.001, *η^2^* =.24], with higher P150 amplitude overall in frontal regions and the left hemisphere. No interaction terms were observed. The between-group relationship was not significant [*p*=.17]. However, when trial numbers were introduced as a covariate, these effects no longer reached significance [all *p*s >.06].

In Figure S2, we depict the ERPs in the frontal ROI (right and left hemisphere), where a P150 component was higher amplitude than the tempo-parietal regions, irrespective of order of presentation of the stimulus. Within the same area of interest, there were no significant differences between the standards for either the LR or the high-risk groups. Note that in the plots below, the response often begins before time zero. Although baseline correction has been applied, this is an issue often encountered with short inter-trial intervals where responses from the previous stimulus are not returning to the baseline. This is not an issue for gamma analysis described in the main text, which returns to the baseline at a quicker rate.


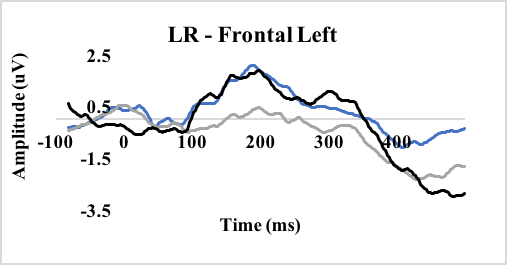

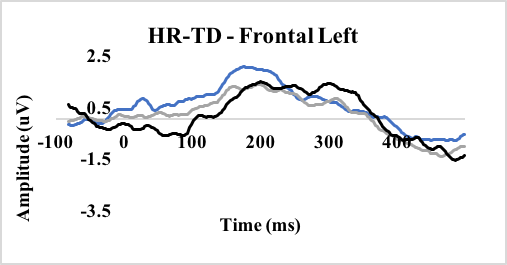

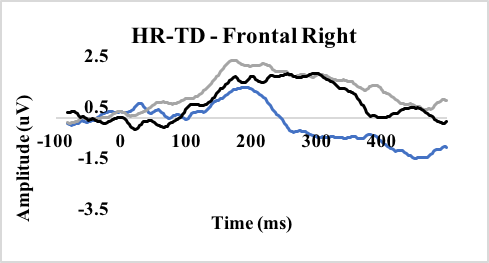

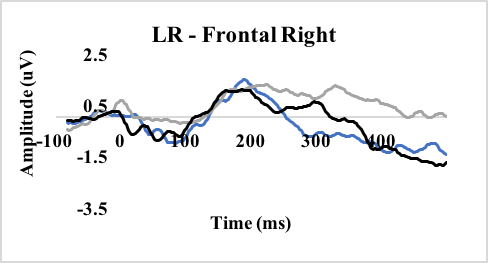

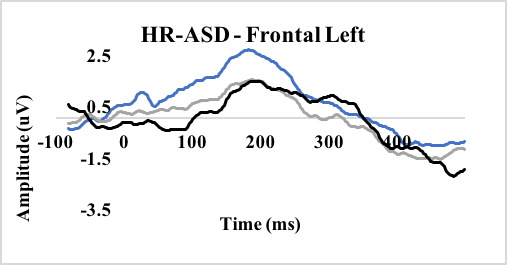

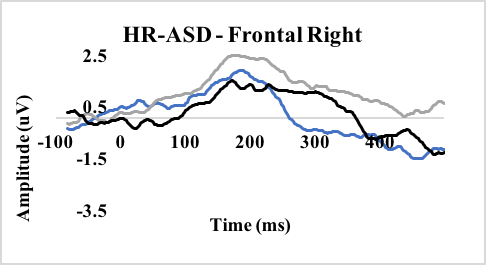

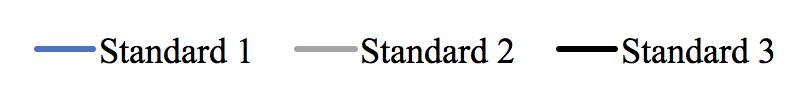


***Figure S2*** – Event-Related Potential (ERP) figures for the three repetitions of the Standard tone in the right and left frontal clusters, where a significant P150 difference was observed relative to the tempo-parietal region. No significant differences were observed between the first and third repetition of the Standard, nor between risk groups.

**SM3. Ocular Artefacts Analysis**

The effect of ocular artefacts on the measures used in the present study were examined based on a recently published analysis (9). To eliminate the possibility of that the observed gamma response to tone repetition were due to co-occurring micro saccades (MS) of the eyes, the bipolar horizontal EOG signal from the channels closest to the eye area (channel 32 subtracted from channel 1) was compared with gamma repetition suppression. Each outcome group was analysed separately due to the possibility of atypical MS in ASD relatively to the typically-developing controls (10). The 40-60Hz evoked responses were compared between the change between 1^st^ and 3^rd^ standard in the right tempo-parietal scalp region and the difference in EOG signal between these two stimuli. The correlation was non-significant for LR [*r*(13)=.266, *p*=.358], HR-TD [*r*(43)=-.005, *p=*.975], and ASD [*r*(12)=.148, *p*=.629] groups. Pearson’s product moment correlation showed no association between these two variables [*r*(71)=-.054, *p*=.653] across the three groups combined (see S3).


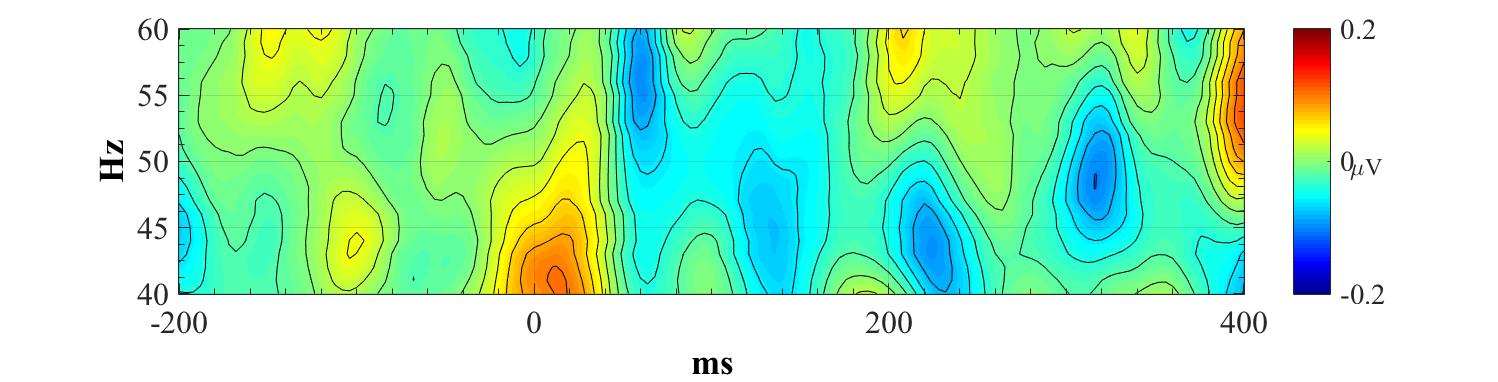

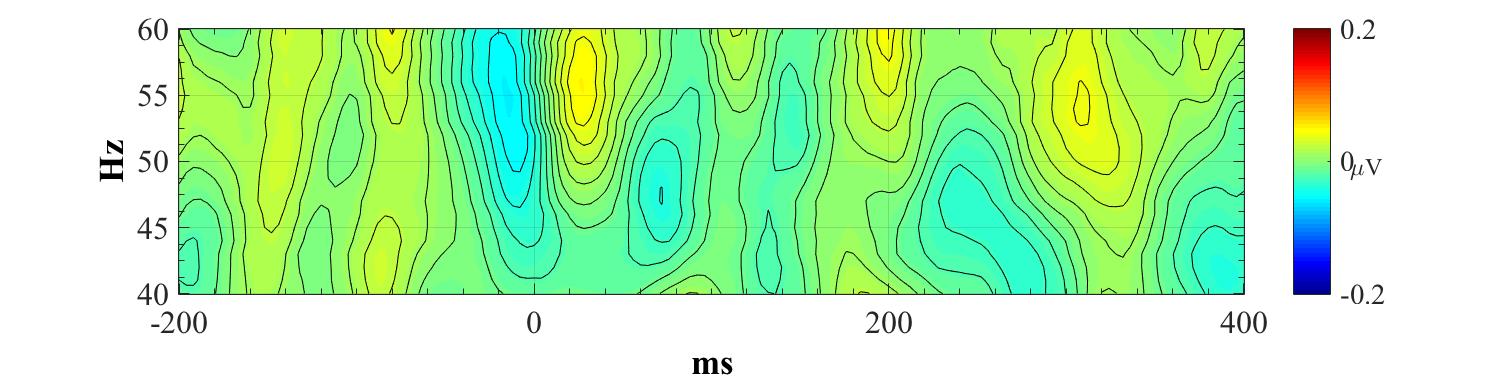

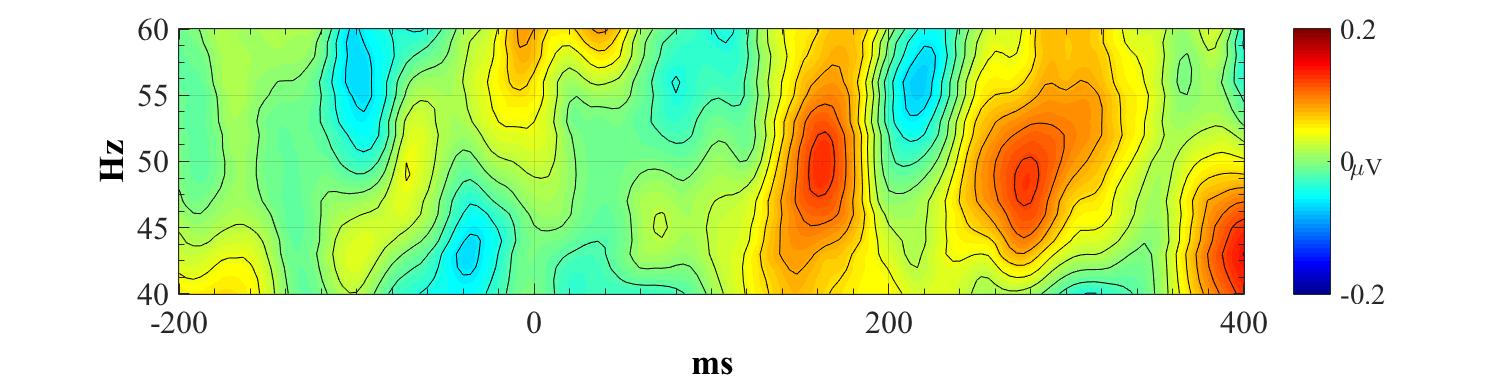

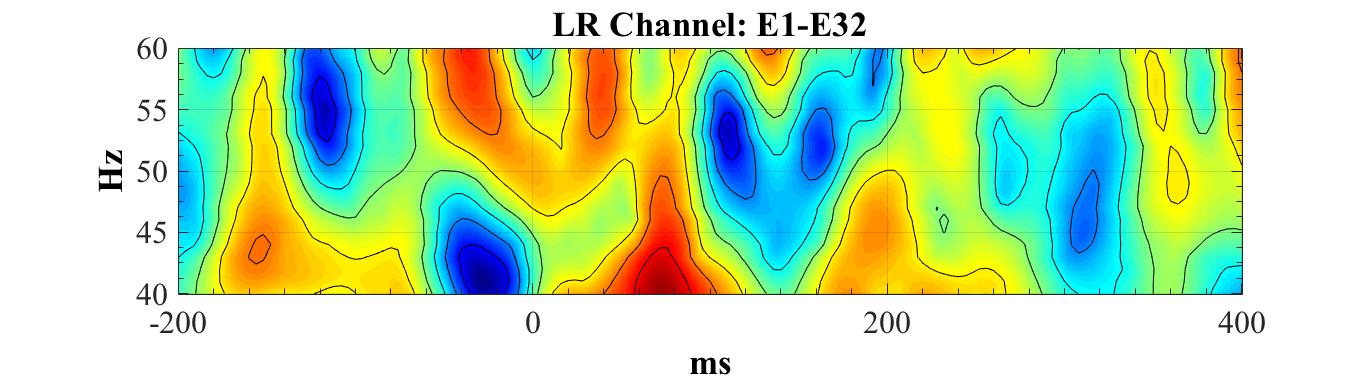

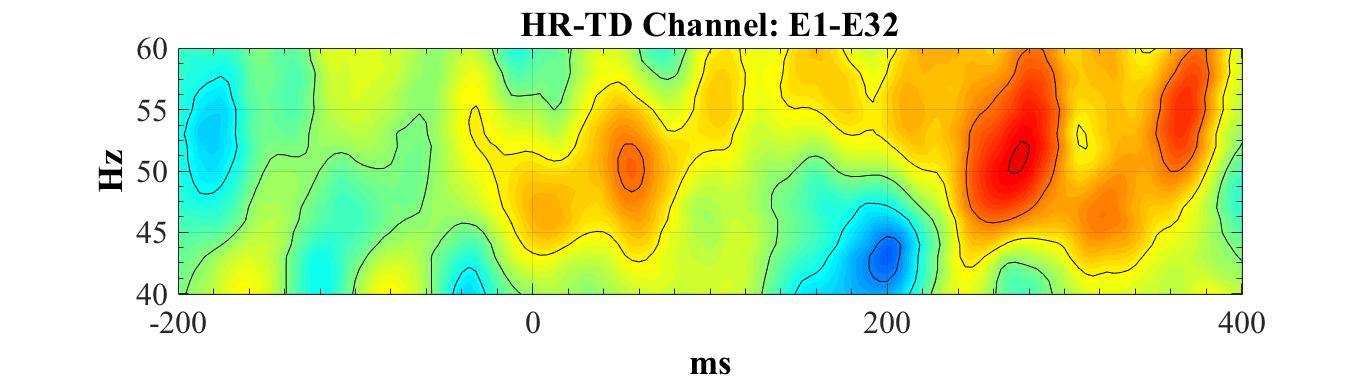

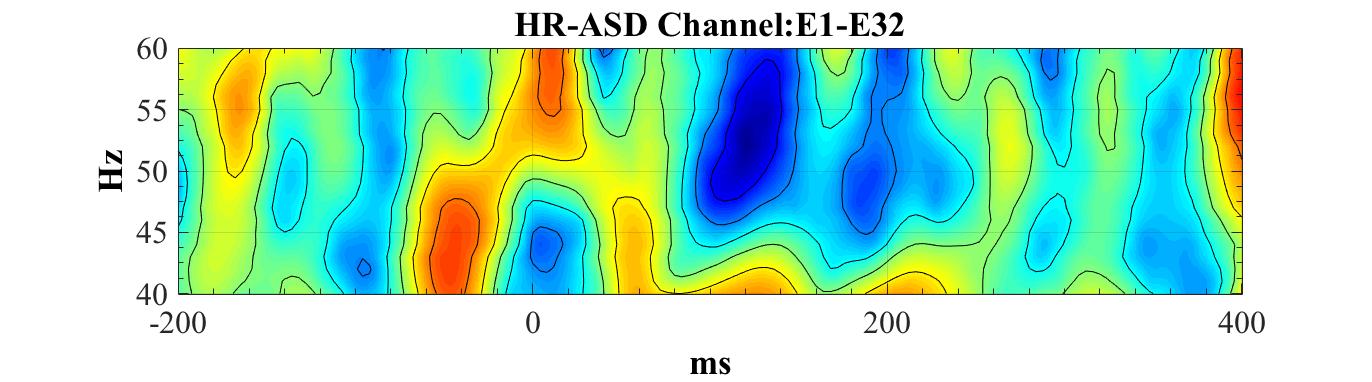

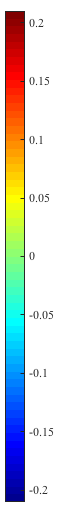


**LR**

**HR-TD**

**HR-ASD**

*Figure S3* – MS analysis found no significant association between (left) the difference between 3^rd^ and 1^st^ repetition of the standard tone and (right) differences in the eye channel data (channel 32 subtracted from channel 1; Standard 3 – Standard 1). Dashed rectangles mark the window of interest.

Further, MS effects were assessed for ITC values by comparing the phase-locking values to the 3rd Standard (where a difference emerged between HR-TD and HR-ASD groups) to EOG signal for that stimulus. No significant association was revealed for LR [r(10)=.488, p=.128], HR-TD [r(40)=.083, p=.606], or HR-ASD [r(12)=.291, p=358] groups. The association remained non-significant when comparing ITC values and EOG signal for the 3rd standard across the three groups [r(63)=.171, p=.178].

**SM4. Additional time-frequency analyses**

***SM4.1. Evoked theta-band comparisons***

Based on existent literature and comments from reviewers, we supplement our analysis of high frequency oscillations in the theta range in the same electrode cites chosen for main analysis. Statistical analyses were conducted on individual averages of time-frequency matrixes from (3-6Hz) over 50-400ms (long range based on differences in latency of low-frequency oscillations, 11). Based on our earlier model, the region of significance for theta band activation was first isolated for the LR group. A paired samples t-test was carried out to look for significant reductions in theta amplitude between Standard 1 and 3 in the frontal regions. The t-tests showed no significant differences between stimuli in the frontal regions [all *p* values >.05]. Analysis over the tempo-parietal regions returned a significant decrease in mean theta amplitude over the right tempo-parietal region [*t*(12)= -5.64, *p*<.001, *η^2^*=.74], but not the left [*t*(12)=-0.53, *p*=.959, *η^2^*=.02], corresponding to the findings of auditory repetition suppression described in the main text.

Then, we investigated differences in repetition suppression response in the right tempo-parietal region in the high-risk groups, with and without autism outcome. For this, we computed a difference score (Standard 3-Standard 1). A one-way ANOVA revealed no significant effect of outcome group [*F*(1,55)=1.03, *p*=.31, *η^2^*=.23], also when co-varied with trial number [*F*(1,53)=1.21, *p*=.27, *η^2^*=.22]. It should be noted that while the mean scores are slightly higher for the HR-ASD group, the confidence intervals overlap and cross the zero mark [HR-TD *M*= *-.*0001*, CI*[-.052 .052]*;* HR-ASD *M= .*055*. CI*[-.061 .172]], see Figure (S4A and B). This finding is in accordance to previous investigations with auditory theta activity, which examined evoked theta activation to auditory stimuli (11,12).

**A**

***Error bars +/- 2 S.E.***


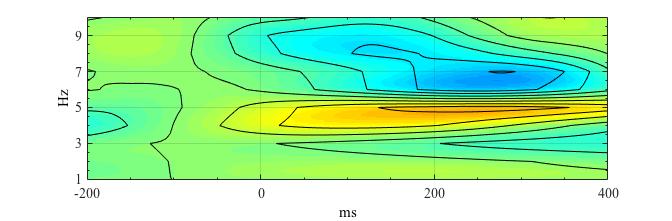

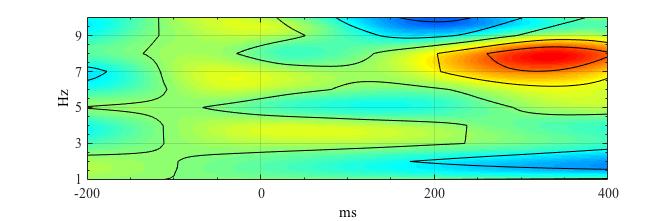

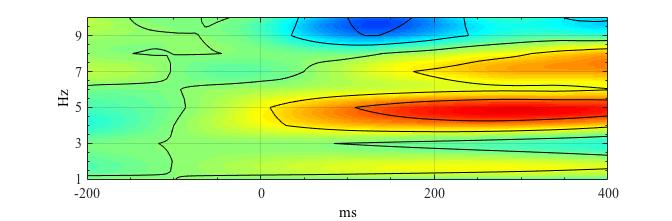

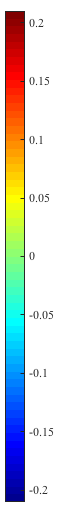


**LR**

**HR-TD**

**HR-ASD**

**B**

*Figure S4* – (A) Mean amplitude difference between 50-400ms in the theta range both evoked and induced activation in the right tempo-parietal ROI. (B) Spectral plots of evoked theta responses (3-6Hz) expressed as the difference between the first and third repetition of the standard tone. *Note that the theta bands and time regions of interest were set prior to visualization or statistical analysis.*

***SM4.2. Late evoked gamma comparisons***

Further, we extracted late gamma amplitude in the 40-60Hz frequency band over 200-350ms in the right tempo-parietal region (see Figure 1 in the main text). This was not featured in the main text as these late differences were not in the priori hypothesis and were only revealed once the group analysis was visualised for the HR-ASD group. We repeat the analysis on a model which previously revealed to be significant in LR and HR-ASD vs. HR-TD groups. An ANOVA showed no significant effect of outcome [*F*(1,55)=.658, *p*=.421, *η^2^*=.012], nor when trial numbers were included [*F*(1,53)=1.21, *p*=.27, *η^2^*=.22]. Although the average plot appears to show strong evoked response in the late time region, there is large variance within group means which could explain our result [*HR-TD M=.*003*; CI*[-.024 .031], *HR-ASD M=.*026*, CI*[-.022 0.73]].

**SM5. Phase-locking (ITC) analysis**

**SM5.1. ITC analysis alpha-beta band**

In addition to changes in amplitude, we examined phase synchronisation through Inter-Trial coherence (ITC). This is derived from computing the consistency in phase-locking of oscillatory signals across trials, and reflects the temporal and spectral synchronisation within the signal. It can provide us with a more direct measure of cortical synchrony, which is not possible with evoked responses. It has been previously shown that ITC values increase with development (13,14). Recent studies have also extracted this measure in auditory processing studies in the theta and gamma band ranges to supplement their analyses (15,16), although it seems that the responses are strongest in the lower frequency ranges (11).

We looked at the aggregated grand average phase-locked response for all infants in the sample within the alpha-beta range in the right tempo-parietal area. Figure 2D in the main text shows a phase-locked response around 100-180ms across 10-20Hz range. Collapsing all standards together further allowed us to increase number of trials which is important to stabilise the response (17). Of note, the amplitude of ITC responses is relatively low (*Mean*=0.071, *Range* 0.026-0.18). Typical ranges for previous studies report comparable values with 6-month-old infants as 0.005 to 0.25 (15) and older children as 0.05 to 0.3 (18), 0 to 0.3 (19), and 0.1 to 5 (20). Between 6 and 12 months, Ortiz-Mantilla and colleagues observed smaller and faster phase synchrony and reduced power (16), which is comparable with ERP literature as they also get stronger with age. Further work will be required to examine age-related change in strength and localization of phase locking to non-linguistic sounds, specifically isolating the age where we begin to see adult-like ITC responses.

**SM5.2. ITC analysis theta band**

We also looked at the aggregated grand average phase-locked response for all infants in the sample within the theta range based on previous investigations of infant phase coherence in auditory processing studies. Theta activity may provide insight into higher order functions such as language specialization (11,21), and has been found a more robust response than ITC in the gamma range (11). It was therefore extracted as a *post hoc* measure. Figure S5 below shows a phase-locked response around 100-180ms in the 3-6Hz range. Statistical comparisons did not reveal any group differences in phase-locking in the theta range [*F(1,55)=.400, p=.53, η^2^*=.007], and this was maintained with trial numbers as a covariate [*F(1,54)=.393, p=.53, η^2^*=.007]. This analysis suggests that although a strong response can be seen in figure S5A and S5B, group differences were apparent in the alpha-beta range.

**
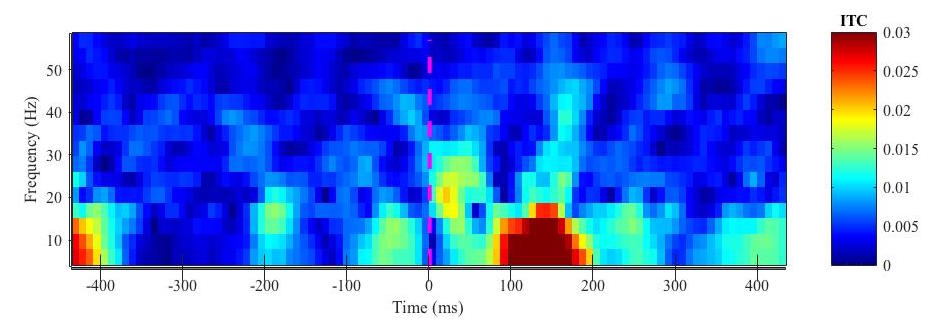
**

*Figure S5 -* Total ITC responses collapsed across all infants and all Standard trials. For statistical comparisons of phase locking in the theta range 100-180ms time-window over 3-6Hz was chosen.

**SM6. Analysis including HR-Atyp group**

We then ran further analyses to understand responses in the HR-Atyp group. A univariate ANOVA was run to look at the differences in 40-60Hz evoked gamma amplitude between Standard 3 and Standard 1. The difference was significant across groups [*F*(2, 74) = 4.18, *p* = .019, *η^2^*=.102], and this remained significant after co-varying trial numbers [*F*(2,74) = 4.21, *p* = .019, *η^2^*=.104]. Specifically, the HR-Atyp group showed a decrease in gamma activation between the 1^st^ and 3^rd^ repetition (similar to HR-TD), while the HR-ASD group showed an increase. Pairwise comparisons show that this difference was significantly higher in HR-ASD infants than HR-TD [*p=*.012] and HR-Atyp [*p*=.008] groups (see Figure S6).

*Figure S6*– Amplitude difference of 40-60Hz evoked gamma in the right tempo-parietal electrodes between Standard 3 and Standard 1for all four groups in the sample. LR group included for reference of a typical repetition suppression response, but not included in the statistical comparison.


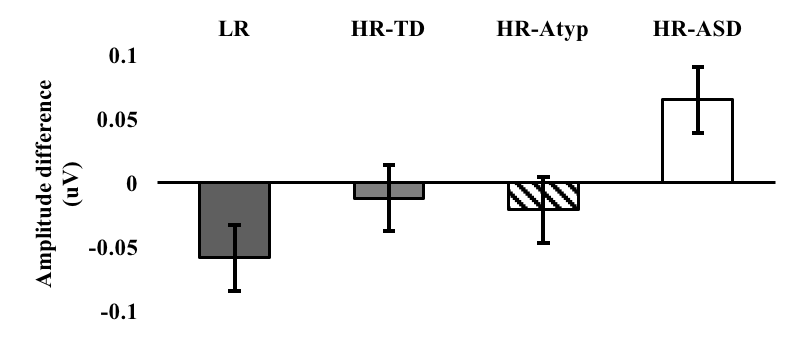


***Error bars +/- 2 S.E.***

Next, the HR-Atyp group was included in the ITC analysis. The ANOVA did not return significant ITC differences between the three high risk groups (TD, Atyp, ASD), [*p*=.157; *p*=.179 with trial numbers co-varied]. It can be seen on the graph below that the HR-Atyp group had higher ITC relative to high-risk siblings who were typically developing (Figure S7), however, future experiments will need to incorporate high trial numbers to explore this possibility.

Lastly, the HR-Atyp group was included in analysis of the composite cortical hyper-reactivity scores, where higher scores on the scale indicated diminished auditory repetition suppression (Figure 2A main text). The ANOVA revealed a significant main effect of group when all HR groups were included [*F*(3,85) = 3.68, *p* = .015, *η^2^*=.115], and also when number of trials is co-varied [*F*(3,84) = 3.64, *p* = .016, *η^2^*=.115]. Pairwise comparisons showed that infants with later typical development had significantly lower cortical hyper-reactivity scores relative to HR-ASD group [*p*=.002], with and without trial numbers as a co-variate.


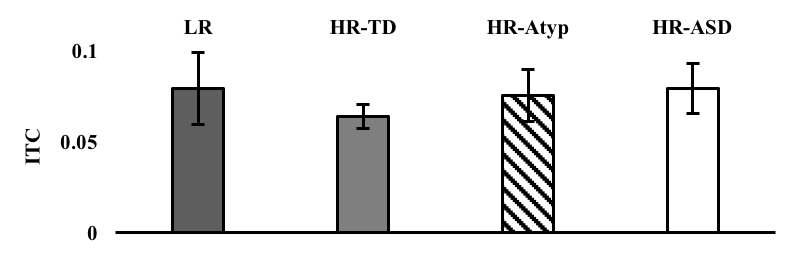


***Error bars +/- 2 S.E.***

*Figure S7* – Intertrial coherence for all groups for all standards collapsed. The group effect remained after HR-Atyp group was added. LR group included for reference.

**References:**

1. Guiraud JA, Kushnerenko E, Tomalski P, Davies K, Ribeiro H, Johnson MH. Differential habituation to repeated sounds in infants at high risk for autism. NeuroReport. 2011 Sep;1.

2. Lord C, Risi S, Lambrecht L, Cook EH, Leventhal BL, DiLavore PC, et al. The Autism Diagnostic Observation Schedule—Generic: A Standard Measure of Social and Communication Deficits Associated with the Spectrum of Autism. J Autism Dev Disord. 2000 Jun 1;30(3):205–23.

3. Gotham K, Pickles A, Lord C. Standardizing ADOS scores for a measure of severity in autism spectrum disorders. J Autism Dev Disord. 2009;39(5):693–705.

4. Rutter M, Le Couteur A, Lord C. Autism diagnostic interview-revised. Los Angel CA West Psychol Serv. 2003;29:30.

5. Rutter M, Bailey A, Lord C. The social communication questionnaire: Manual. Western Psychological Services; 2003.

6. Mullen EM. Mullen scales of early learning. AGS Circle Pines, MN; 1995.

7. Sparrow SS. Vineland Adaptive Behavior Scales. In: Kreutzer JS, DeLuca J, Caplan B, editors. Encyclopedia of Clinical Neuropsychology [Internet]. Springer New York; 2011 [cited 2017 Mar 17]. p. 2618–21. Available from: http://link.springer.com/referenceworkentry/10.1007/978-0-387-79948-3_1602

8. Seery A, Tager-Flusberg H, Nelson CA. Event-related potentials to repeated speech in 9-month-old infants at risk for autism spectrum disorder. J Neurodev Disord [Internet]. 2014 [cited 2017 May 23];6(1). Available from: https://www.ncbi.nlm.nih.gov/pmc/articles/PMC4416338/

9. Kampis D, Parise E, Csibra G, Kovács ÁM. On potential ocular artefacts in infant electroencephalogram: a reply to comments by Köster. Proc R Soc B. 2016 Jul 27;283(1835):20161285.

10. Schmitt LM, Cook EH, Sweeney JA, Mosconi MW. Saccadic eye movement abnormalities in autism spectrum disorder indicate dysfunctions in cerebellum and brainstem. Mol Autism [Internet]. 2014 Sep 16 [cited 2017 Feb 14];5. Available from: http://www.ncbi.nlm.nih.gov/pmc/articles/PMC4233053/

11. Musacchia G, Ortiz-Mantilla S, Choudhury N, Realpe-Bonilla T, Roesler C, Benasich AA. Active auditory experience in infancy promotes brain plasticity in Theta and Gamma oscillations. Dev Cogn Neurosci. 2017 Aug 1;26:9–19.

12. Rojas DC, Wilson LB. Gamma-band abnormalities as markers of autism spectrum disorders. Biomark Med. 2014 Mar;8(3):353–68.

13. Bishop DVM, Anderson M, Reid C, Fox AM. Auditory development between 7 and 11 years: an event-related potential (ERP) study. PloS One. 2011 May 9;6(5):e18993.

14. Muller V, Gruber W, Klimesch W, Lindenberg U. Lifespan differences in cortical dynamics of auditory perception. Dev Sci. 2009 Nov;12(6):839–53.

15. Ortiz-Mantilla S, Hämäläinen JA, Musacchia G, Benasich AA. Enhancement of Gamma Oscillations Indicates Preferential Processing of Native over Foreign Phonemic Contrasts in Infants. J Neurosci. 2013 Nov 27;33(48):18746–54.

16. Ortiz-Mantilla S, Hämäläinen JA, Realpe-Bonilla T, Benasich AA. Oscillatory Dynamics Underlying Perceptual Narrowing of Native Phoneme Mapping from 6 to 12 Months of Age. J Neurosci. 2016 Nov 30;36(48):12095–105.

17. Cohen MX. Analyzing Neural Time Series Data: Theory and Practice. MIT Press; 2014. 615 p.

18. Edgar JC, Fisk CL, Liu S, Pandey J, Herrington JD, Schultz RT, et al. Translating Adult Electrophysiology Findings to Younger Patient Populations: Difficulty Measuring 40-Hz Auditory Steady-State Responses in Typically Developing Children and Children with Autism Spectrum Disorder. Dev Neurosci. 2016;38(1):1–14.

19. Bishop DVM, Hardiman MJ, Barry JG. Is auditory discrimination mature by middle childhood? A study using time-frequency analysis of mismatch responses from 7 years to adulthood. Dev Sci. 2011 Mar 1;14(2):402–16.

20. Nash-Kille A, Sharma A. Inter-trial coherence as a marker of cortical phase synchrony in children with sensorineural hearing loss and auditory neuropathy spectrum disorder fitted with hearing aids and cochlear implants. Clin Neurophysiol Off J Int Fed Clin Neurophysiol. 2014 Jul;125(7):1459–70.

21. Bosseler AN, Taulu S, Pihko E, Mäkelä JP, Imada T, Ahonen A, et al. Theta brain rhythms index perceptual narrowing in infant speech perception. Front Psychol [Internet]. 2013 Oct 11 [cited 2018 Mar 17];4. Available from: https://www.ncbi.nlm.nih.gov/pmc/articles/PMC3795304/
